# Lattice and spin dynamics in multiferroic BiFeO_3_ and *R*MnO_3_

**DOI:** 10.1093/nsr/nwz055

**Published:** 2019-05-02

**Authors:** Yan Song, Ben Xu, Ce-Wen Nan

**Affiliations:** School of Materials Science and Engineering, and State Key Laboratory of New Ceramics and Fine Processing, Tsinghua University, Beijing 100084, China

**Keywords:** multiferroics, phonon, magnon

## Abstract

The multiferroic materials BiFeO_3_ and *R*MnO_3_ exhibit coexisting magnetic order and ferroelectricity, and provide exciting platforms for new physics and potentially novel devices, where intriguing interplay between phonons and magnons exists. In this review, we paint a complete picture of bulk BiFeO_3_ together with orthorhombic and hexagonal *R*MnO_3_ (*R* includes rare-earth elements and yttrium) by summarizing the dynamics of spin and lattice and their magnetoelectric coupling, as well as the methods of controlling these characteristics under non-equilibrium conditions, from experimental and simulation perspectives.

## INTRODUCTION

The advancement beyond complementary metal-oxide-semiconductor devices demands materials with strong logic state stability and high switching efficiency [1,2]. Multiferroic materials are intrinsically promising candidates in this regard because their low energy dissipation in switching and high energy efficiency form a barrier to stabilize the order parameter [2]. However, it is still challenging to overcome the precession timescale limitation and switch the multiferroic antiferromagnetic (AFM) state with a high stability of 100*k*_B_*T* locally on the nanoscale. The recently demonstrated possibility of direct pumping of spin excitations in AFM materials with the help of a freely propagating terahertz (THz) wave suggests that such waves are strongly coupled to the excitations in the medium and provide a useful pathway for manipulating the material structure and properties to overcome the abovementioned limitation. In these processes, the coupling between the lattice and spin excitations is essential; however, this topic remains highly underrated and not sufficiently understood.

**Figure 1.**
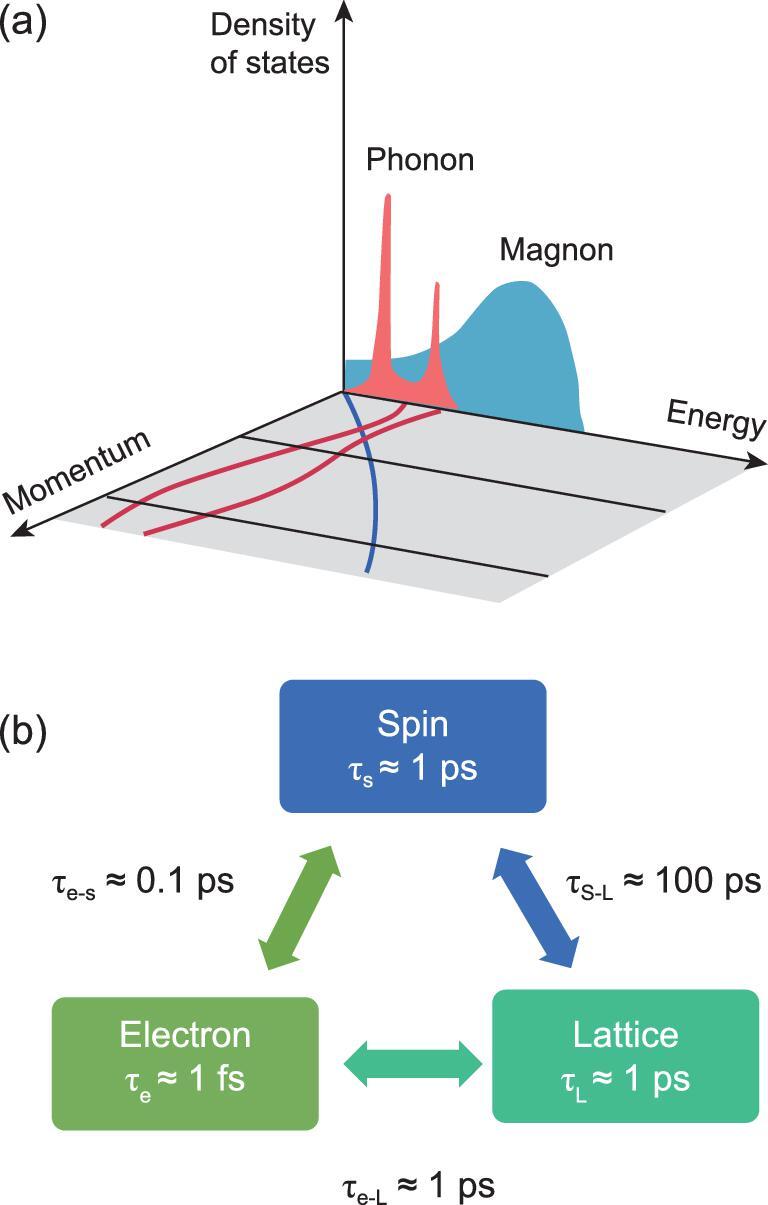
(a) Energy–momentum relation of phonons and magnons in complex solids. (b) Schematic of the three subsystems with their characteristic relaxation times and coupling parameters.

The collective lattice and spin excitations are addressed as quantized waves called phonons and magnons. Both phonons and magnons obey boson behavior and therefore follow Bose–Einstein statistics [3]. These quasiparticles can be completely described by their dispersion relations between energy (frequency) and wave vector, which is important for understanding atomic bonding, to determine the underlying interactions governing the spin dynamics and obtain detailed information about complex spin structures (Fig. 1) [4]. Strong anharmonicity exists in multiferroics and allows higher-order zone-center magnons to become dipole active, i.e. to become other quantized excitations unique in multiferroics as electromagnons [5,6].

BiFeO_3_ (BFO) and *R*MnO_3_ (*R* includes rare-earth elements and yttrium) are the two most widely studied multiferroics. BFO and hexagonal (*h*)-*R*MnO_3_ are type-I multiferroics whose ferroelectric (FE) transition temperatures are well above their magnetic ones. Meanwhile, orthorhombic (*o*)-*R*MnO_3_ (e.g. TbMnO_3_, DyMnO_3_ and (Tb, Gd)MnO_3_) is a type-II multiferroic with strong magnetoelectric (ME) coupling, and the ordered phases occur at very low temperatures. It is worth mentioning that not all *o*-*R*MnO_3_ are multiferroics, e.g. manganites with *R* = Nd, Sm, Eu, Ho are not multiferroics. The remainder of this review is divided into two main parts: firstly, we focus on experimental investigations as well as the manipulation of phonons, magnons, electromagnons, and the coupling between them in BFO and *R*MnO_3_; then, we address the simulations and pre-diction schemes used to demonstrate and manipulate the dynamics of phonons and magnons. Finally, we summarize the conclusions.

## EXPERIMENTS

At present, experimental measurements of phonons and magnons are performed using two main types of methods: inelastic neutron scattering (INS) and optical techniques, where Raman, infrared (IR), and THz spectroscopy are most commonly used; these have different selection rules and spectral weights [7]. INS could be used to measure the dispersion relations in full momentum space, whereas optical means are restricted to near the Brillouin zone (BZ) center. However, the energy resolution of INS in the low-energy region is rather poor, while THz spectroscopy exhibits superiority in this region. INS measures phonon and magnon spectra in the whole BZ including electromagnons, but hybrid excitations can only be determined by IR or THz spectra based on full polarization analysis, observation of directional dichroism or transfer of oscillator strength of some polar phonon to spin excitation [8,9].

### Phonons

The vibrational spectra of BFO [8,10–17], *o*-*R*MnO_3_ [18], and *h*-*R*MnO_3_ [7,19–23] have been well characterized. All of them converge to a common phonon picture reflecting the symmetry of the crystals. The symmetry of BFO is rhombohedral R3c [11], and P6_3_cm and P6_3_/mmc for the FE and paraelectric states of *h*-YMnO_3_ [7]. Note that usually fewer phonons are experimentally observed than that are allowed by symmetry, because some of the modes are overlapping or have low intensities. The phonon symmetry needs to be correctly assigned using polarized Raman techniques [12,14,24] and infrared spectroscopy [8,17,18,22]. This symmetry information of phonons is crucial for understanding the nature of phase transitions and identifying connections between physical properties and atomic motions [25–27].

As the temperature increases, the energy of particular phonon modes decreases because of bond softening [10,11], and some of them disappear due to the crystal structure change above FE transition. Moreover, the phonon density of states measured by INS shows broadening of the entire phonon spectrum, indicating strong anharmonicity due to phonon–phonon interactions [28]. Hybridization or coupling also occurs between different branches [29].

### Magnons

In type-I multiferroics, since the FE transition takes place at higher temperatures than the magnetic ordering, unperturbed magnon (phonon) dispersion can be measured below (above) *T*_N_. The non-spin-flip data capture the phonon signal, while the spin-flip signal is purely magnetic.

The zone-center frequencies of magnons are normally below those of phonons, with high-energy spin dynamics for super-exchange interaction including the nearest neighbor (NN) and next-nearest neighbor (NNN) exchanges, and low-energy spin dynamics for inter-layer coupling and single-ion anisotropy (SIA) [6], where discrepancies still exist between the simulation and dispersion curves obtained experimentally [30]. The ratio between the NN and NNN exchanges determines the local AFM order, and the Dzyaloshinskii–Moriya interaction (DMI) stabilizes a long-period spin cycloid in BFO.

**Figure 2.**
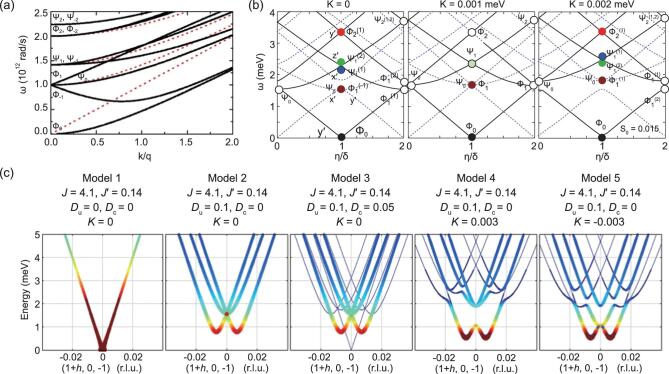
(a) Theoretical result of the magnon spectrum for a cycloid without the anisotropy term. The perpendicular component to the cycloid plane and parallel component along the polarization direction are depicted by the solid and dashed lines, respectively. The Φ_n_ (cyclon) and Ψ_n_ (extra-cyclon) are the in-plane and out-of-plane modes, respectively. (b) Magnon modes calculated by the full spin Hamiltonian. All possible excitations are showed by dashed lines. (c) Calculated magnon dispersion along [h 0 0]_hex_ near (1, 0, -1)_hex_ for several models described in the text [4]. Copyright 2019, IOP Publishing.

In BFO, spin cycloids excite two categories of magnons corresponding to spin-wave excitations: cyclons (denoted Φ, in the cycloid plane) and extra-cyclons (or spin-flip, denoted Ψ, out of the cycloid plane), which can be demonstrated using Raman spectroscopy [6], THz spectroscopy [5,8,31,32] and millimeter-wave and IR spectroscopy [15]. The peak locations of the Φ modes are equally spaced starting at zero frequency, while those in the Ψ sequence are not regularly spaced at low frequencies, in agreement with the theoretical prediction [33]. Different DMIs and SIAs can only introduce significant changes at the zone center at lower energies (shown in Fig. 2) [4]. In TbMnO_3_, three modes exist corresponding to the rotation of both the spin plane and polarization direction about the *z* axis; rotation of the spin plane about the *x* axis, which is not coupled to the polarization; and the sliding mode, i.e. phason, of the spiral [34], and have been observed in INS [35] and THz studies [36–41]. Moreover, multiple excitation modes such as two-magnon modes have been observed in IR reflectivity spectra of TbMnO_3_ [9].

In *h*-*R*MnO_3_, such as YMnO_3_, the spins on Mn^3+^ ions are ordered antiferromagnetically and frustrated strongly on triangles in the *ab* plane with a 120° angle. Magnons were investigated using IR, THz and Raman spectroscopies [7,42]. Excitation at the zone boundary in the *ab* plane and linear field-induced magnon dispersion splitting close to the zone center [43] suggest a possible 3D magnetic ground state of the system. However, discrepancies still exist between INS dispersion curves and theoretical calculations of the exchange parameter ratio *J*_NN_/*J*_NNN_, which implies that the standard interpretation of magnon spectra needs to be revised and that magnon–phonon and magnon–magnon interactions need to be included.

When increasing the temperature or magnetic field, softening and broadening occur in the spin-wave spectrum [28], which reflects the frustrating NNN interactions, weakened NN interactions, and strong anharmonicity of the magnon–magnon and magnon–phonon interactions. Moreover, non-monotonic behavior of the INS scattering intensity has been observed with increasing temperature [44]. Broadening of the magnon spectrum and excitation splitting have been found [8,45], although they require further study [35].

### Phonon–magnon coupling and electromagnons

Generally, lattice and spin coupling manifest themselves as anomalous temperature dependence of the peak position deviating from standard anharmonicity-related phonon decay and anomalous hardening below *T*_N_ [10,23,46]. When the phonon–magnon coupling becomes sufficiently strong, a complex magnetic phase can be introduced; e.g. a longitudinal spin-density wave and spiral phase can exist at different temperatures in TbMnO_3_ [9]. Magnon–phonon coupling is so strong that it governs the polarization dependence of magnon absorption in BFO [47]. Most importantly, the transverse acoustic phonons measured at the zone boundary clearly broaden in energy when *T*_N_ is approached from below, indicating that the acoustic phonon modes and magnetic order are coupled in this multiferroic material [29].

A quasiparticle called an electromagnon (EM) [48–51] can be excited and is a hybrid of magnon- and phonon-like modes, which can be excited by the electric and magnetic fields of a photon, respectively. The activation of these excitations can stem from either DM interaction [33,52–55], or exchange striction [37,56,57]. In addition to mixing the modes, the ME coupling shifts the frequencies with respect to the bare magnon or phonon frequencies. However, these energy shifts are small [33,52] in BFO, so the magnetic resonances and electromagnons are coincident within the experimental resolution. Depending on the magnetic modulation and anharmonicity of the material, EM with higher quantum number can appear, which can be attributed to Umklapp coupling between magnons and phonons [33,52] as well as non-zero SIA, where the latter can also split Ψ_n_ and Φ_n_ to Ψ_±n_ and Φ_±n_ [6,45], as shown by THz spectroscopy [5] of BFO, *o*-*R*MnO_3_ [9,38–40]. However, it is still under debate why optical phonons and electromagnons that are separated by such a large energy scale can experience direct coupling.

In *h*-*R*MnO_3_, the FE and magnetic orderings are no longer concomitant since the electric polarization appears far above room temperature, while strong magnon–phonon coupling still exists. One of the consequences is that extra hardening deviation occurs below *T*_N_ for several phonon branches [20,22,23,46]. At the same time, strong deviations from linear spin-wave theory excluding magnon–phonon coupling have been observed in magnon dispersion curves [41]. Consequently, the lower mode at the BZ boundary moves downwards in energy and an additional mode at high energy appears in the phonon spectrum of *h*-*R*MnO_3_. Moreover, avoided crossing (anti-crossing) at the zone boundary in polarized INS was revealed and exhibited mixed magnon–phonon characteristics [7,19,43,58]. Furthermore, spontaneous magnon decay, i.e. finite magnon lifetime, occurs because of the anharmonic higher-order terms caused by non-collinear spin structures [41].

### Manipulation of phonons and magnons

Magnetic states can be manipulated either by switching the helicity of AFM-order spin cycloids in TbMnO_3_ using a continuous-wave laser beam with an energy of 2.3 eV [59] or by tuning the motion of the magnetization vector to follow an arbitrarily designed direction and amplitude of polarization in NiO [60]. The latter method is performed by applying two linearly polarized laser pulses with a properly tuned azimuthal angle *ψ* and time delay *τ* [60]. A similar idea was later implemented in AFM three-sublattice ordering of the magnetic Mn^3+^ moments in hexagonal YMnO_3_, where full 3D magnetization control was realized by a pair of time-delayed polarization-twisted femtosecond laser pulses [61]; thereby, it is possible to store multiple pieces of information.

## SIMULATIONS

The following section will concentrate on theories and simulation techniques used to identify the magnetic interactions in multiferroic materials; to demonstrate the dispersion curves of phonons, magnons, and electromagnons; and lastly to manipulate the magnetic states based on the strong non-linear phonon–magnon coupling.

### Hamiltonian for multiferroic systems

The total Hamiltonian for multiferroic systems has been thoroughly reviewed in the previous literature [62] and can be expressed in several terms as ${{\cal H}_{{\rm{exch}}}} + {{\cal H}_{{\rm{DM}}}} + {{\cal H}_{{\rm{SIA}}}}$, with the NN exchange ${J_{i\!j}}$, NNN exchange $J_{ij}^{\rm{^{\prime}}}$, DMI *D_ij_*, and SIA *K*_SIA_. The exchange interaction ${{\cal H}_{{\rm{exch}}}}$ can be expressed as

(1) \begin{eqnarray*} {{\cal H}_{{\rm{exch}}}} &=& \mathop \sum \limits_{r,i,j} J_{ij}^*{S_i} \cdot {S_j}{\rm{\ }} = \mathop \sum \limits_{r,{\rm{NN}}} {J_{ij}}{S_i} \cdot {S_{{\rm{NN}}}}{\rm{\ }} \nonumber\\ &&+\, \mathop \sum \limits_{r,{\rm{NNN}}} J_{ij}^{\rm{^{\prime}}}{S_i} \cdot {S_{{\rm{NNN}}}}. \end{eqnarray*}

If *J_ij_* > 0, the exchange interaction favors the AFM state, while, if *J_ij_* < 0, it favors the ferromagnetic state. The DMI term ${{\cal H}_{{\rm{DM}}}}$ contributes to the cycloid structure of spins and arises from the interplay between broken inversion symmetry and spin–orbit coupling [63,64], where

(2) \begin{equation*} {{\cal H}_{{\rm{DM}}}} = {\rm{\ }} - \mathop \sum \limits_{r,i,j} {D_{ij}} \cdot ( {{S_i} \times {S_j}} ). \end{equation*}

The DMI favors perpendicular spin alignment, and the strength is proportional to the rotation angle of the oxygen octahedral. The SIA Hamiltonian is

(3) \begin{equation*} {{\cal H}_{{\rm{SIA}}}} = \ - \mathcal{K}\mathop \sum \limits_r {\left( {{S_r} \cdot \hat{c}} \right)^2} \end{equation*}

For the uniaxial case, $\mathcal{K}$ > 0 corresponds to the easy axis and $\mathcal{K}$ < 0 to the easy plane. SIA comes mainly from anisotropic deformation of the structure as well as the competing FE and antiferrodistortive (AFD) distortions.

The above terms of the Hamiltonian dominate the magnetic behavior of BFO and *h*-*R*MnO_3_, although the DMI can be more specified in different directions. However, for *o*-*R*MnO_3_, the above Hamiltonians cannot precisely describe the complex phase diagram of magnetic states, such as the existence of E-AFM states. The Ising mode [65], two-orbital double-exchange model [66,67], and bond alternation model of FM exchange [68] were proposed, before higher-order coupling terms such as a biquadratic term ${{\cal H}_{{\rm{biq}}}}$ [69] and four-spin ring exchange term ${{\cal H}_{4{\rm{sp}}}}$ [70] were included to predict magnetic structures and important features of spin-wave spectra that cannot be obtained from the Heisenberg Hamiltonian [71–73]:

(4) \begin{equation*} {{\cal H}_{{\rm{biq}}}} = {\rm{\ }} - \mathop \sum \limits_{i,j} {B_{ij}}{( {{S_i} \cdot {S_j}} )^2} \end{equation*}

(5) \begin{eqnarray*} {{\cal H}_{4{\rm{sp}}}} &=& \mathop \sum \limits_{i,j,k,l} {g_{ijkl}}\big[({{S_i} \cdot {S_j}})({{S_k} \cdot {S_j}}) + ({{S_i} \cdot {S_l}})\nonumber\\ && \times\, ({{S_j} \cdot {S_k}}) - ({{S_i} \cdot {S_k}})({{S_j} \cdot {S_l}}) \big]. \end{eqnarray*}

Higher-order exchange couplings can be obtained from the consecutive hopping of electrons between a series of four NN spins and have been invoked to describe the detailed energetics of orthorhombic TbMnO_3_, as in [69,74].

The four-spin ring Hamiltonian fits the energy of *o*-*R*MnO_3_ from DFT [69] and certain features in neutron diffraction patterns [75,76]. Moreover, it not only explains the unexpected polarization direction [76], but also solves the discrepancy between the predicted and observed orders of the electric polarization magnitude of E-AFM-ordered TbMnO_3_ [74,77–79].

### Determination of exchange parameters

For precise evaluation of the above Hamiltonian, it is important to identify the coupling constants accurately [80], which can be done by using first-principles methods to calculate the total energy of four carefully designed collinear spin configurations. *J_ij_*, *D_ij_*, *K*_SIA_, and their derivatives can then be obtained by extracting the linear combination of the energies ${{\cal H}_1},\ {{\cal H}_2},\ {{\cal H}_3},\ {{\cal H}_4}$ of these four different configurations (Eq. (7)), where the forces can be obtained directly from many standardized DFT schemes as Hellmann–Feynman forces [81]. The total energy of the system with collinear spin alignment is calculated when spin states at two sites *i* and *j* within the given unit cell are modified, with the magnetism of the system fully described by the Heisenberg Hamiltonian

(6) \begin{eqnarray*} {{\cal H}_{{\rm{spin}}}} = {J_{ij}}\ {S_i} \cdot {S_j} + {S_i} \cdot {Q_i} + {S_j} \cdot {P_j} + {{\cal H}_{{\rm{other}}}}, \nonumber\\ \end{eqnarray*}

where ${Q_i} = \mathop \sum \limits_{m \ne i,\!j} {J_{im}}{S_m}$, ${P_j} = \mathop \sum \limits_{m \ne i,\!j} {J_{jm}}{S_m}$, and ${{\cal H}_{{\rm{other}}}} = \mathop \sum \limits_{m \ne i,\!j} {J_{mn}}{S_m} \cdot {S_n}$,

(7) \begin{equation*} {J_{ij}} = \frac{{{{\cal H}_1} + {{\cal H}_4} - {{\cal H}_2} - {{\cal H}_3}}}{{4{S^2}}}. \end{equation*}

The derivative of ${J_{ij}}$ with respect to the displacement ${\xi _{k{\rm{\alpha }}}}$ can be found to be

(8) \begin{eqnarray*}\frac{{\partial {J_{ij}}}}{{\partial {\xi _{k{\rm{\alpha }}}}}} = \frac{1}{{4{S^2}}}\ \left( {\frac{{\partial {{\cal H}_1}}}{{\partial {\xi _{k{\rm{\alpha }}}}}} + \frac{{\partial {{\cal H}_4}}}{{\partial {\xi _{k{\rm{\alpha }}}}}} - \frac{{\partial {{\cal H}_2}}}{{\partial {\xi _{k{\rm{\alpha }}}}}} - \frac{{\partial {{\cal H}_3}}}{{\partial {\xi _{k{\rm{\alpha }}}}}}} \right)\!. \nonumber\\ \end{eqnarray*}

Regarding the system with its spin configuration in the non-collinear state, the calculation method remains the same in terms of still using four magnetic ions within a unit cell, while the total energy with respect to the rotation angle *α* away from the initial collinear state is fitted to the form of the Heisenberg model, where the exchange coupling constants *J_ab_* and *J_c_* can be extracted [82]. The problem of identifying coupling constants is essentially one of parameter fitting with certain numbers of equations and unknown constants. Based on this understanding, four spin ring coupling constants were evaluated for *o*-*R*MnO_3_ in an overdetermined system, where the energies of non-equivalent collinear magnetic orders were calculated for a number of states much greater than the number of constants [69]. An array of equations was solved by using the least mean squares method, and all of the abovementioned couplings were found.

### Quasiparticle spectrum

The quantized excitations of the lattice or magnetic subsystem, namely, phonons and magnons, are completely described by their dispersion relation, which is strongly dependent on the atomic bonding and magnetic coupling. Most multiferroic materials exhibit severe modification of the magnetic configuration alongside structural changes, indicating the possibility of strong phonon–magnon coupling.

### Phonons

The starting point of phonon dispersion is correct evaluation of the strength of a particular distortion mode or force constant [83,84]. The former can be readily obtained using shell models [85,86], while the latter needs to be determined from the dynamical matrix of the system [87,88]. In these methods, the total energy is obtained by performing first-principles calculations, using different functionals such as the local density approximation (LDA), generalized gradient approximation (GGA), LDA+*U*, GGA-Wu–Cohen (WC), and Heyd–Scuseria–Ernzerhof functionals [26,89]. Note that although LDA can provide quantitively satisfying agreement with experimental results, some functionals can achieve much better agreement for particular branches, such as the B1-WC functional for the E modes in BFO [90].

Zone-center phonon frequencies of BFO obtained via the above methods fit well most of the peaks in absorption spectra obtained by either Raman or IR measurements [8,16,17,25,26,91,92]. Moreover, through e.g. detailed mode analysis, the transverse optic modes A_1_(TO_1_) and A_1_(TO_2_) were found to involve FE distortion and AFD rotation, respectively, in BFO, which was verified by Ginzburg–Landau calculations [33]. Furthermore, the effects of macroscopic electronic polarization can also be considered by including long-range Coulomb forces in DFT calculations [25,87]. Regarding *R*MnO_3_, discrepancies still exist between the phonon spectra calculated using shell models and experimental measurements, which may be due to over-simplification in the shell model calculations [86]. Indeed, first-principles electronic structure calculations for YMnO_3_ tend to yield higher phonon energies for the low-energy E_1_ modes, compared with those obtained using the shell model [93].

### Magnons

Magnon behavior has been reviewed for BFO [4] and *h*-*R*MnO_3_ [94]. Generally speaking, the high- and low-energy parts of the magnon spectrum are dominated by super-exchange interactions such as *J_ij_* and DMI/SIA, respectively, because DMI/SIA originates from spin–orbit coupling and is much weaker than the exchange interaction. Moreover, SIA will introduce anharmonic effects into a long cycloid or even destroy it if sufficiently strong.

Several methods of calculating magnon spectra to fit the peak positions in IR and Raman spectroscopy or the dispersion curves in full momentum space in INS have been proposed. In the first case, the stochastic Landau–Lifshitz–Gilbert (LLG) equation with fluctuation terms was considered, and peaks in magnetic susceptibility curves were shown to indicate magnons [27]. A similar method was implemented to obtain the magnon dispersion for BFO in the full BZ, starting from the Ginzburg–Landau free-energy expression [33] that includes the contributions of the AFM and FE order parameters *L* and *P*, respectively, as well as the interaction between them, as shown in the following equation:

(9) \begin{eqnarray*}F &=& \frac{{G{L^4}}}{4}{\rm{\ }} + \frac{{A{L^2}}}{2} + \frac{{c\mathop \sum \nolimits_i {{\left( {\nabla {L_i}} \right)}^2}}}{2} \nonumber\\ && -\, \alpha P \cdot \left[ {L\!\left( {\nabla \cdot L} \right) + L \times \left( {\nabla \times L} \right)} \right] \nonumber\\ && -\, P \cdot E + \frac{{r{M^2}}}{2} + \frac{{aP_z^2}}{2} + \frac{{uP_z^4}}{4} \nonumber\\ && +\, \frac{{{a_ \bot }\left( {P_x^2 + P_y^2} \right)}}{2} \end{eqnarray*}

where *L* = |*M*_1_ − *M*_2_| is a Néel vector describing the staggered sublattice magnetization; *M* = |*M*_1_ + *M*_2_| is the total magnetization of the materials; and *P_X_*, *P_Y_*, and *P_Z_* are the magnitudes of the FE polarization along the *x*, *y*, and *z* axes, respectively. Therefore, *L* can be determined from the linearized equations of motion for the FE and AFM order parameters obtained by the variational theorem. Basically, the simple spin waves can be determined from the fluctuations ${\rm{\delta }}{{\bf L}}$, with the cyclon order parameter Φ and out-of-plane order parameter Ψ referring to the phase fluctuation of the cycloid ground state and spin fluctuation out of the cycloid plane (the *xz* plane), respectively:

(10) \begin{eqnarray*} {\rm{\delta }}{{\bf L}} &=& {\rm{\ }}[\phi\! \left( {{\bf r}} \right),\psi\! \left( {{\bf r}} \right),{\rm{\delta }}{{\bf p}}\!\left({{\bf r}} \right)]\ \nonumber\\ &=& \mathop \sum \limits_n \left[ {\phi \!\left({{\bf r}} \right),\psi\! \left( {{\bf r}} \right),{{\boldsymbol{p}}_n}} \right]\ {e^{inqx}}{e^{i{\boldsymbol{k}} \cdot r}}. \nonumber\\ \end{eqnarray*}

Series of parabola-like dispersion curves of magnons were found, with anti-crossing between the optical phonon dispersion and magnon branches at finite *k*, and the frequency was dependent on $q\ = {\rm{\ \alpha }}{P_0}/c$, where *α* and *c* are from the free-energy expression in Eq. (9), and *P*_0_ is the polarization of an easy-axis FE with uniform polarization [33].

The other method of obtaining the full momentum space spectrum starts from the spin Hamiltonian mentioned in the previous section. A second quantization was implemented by using the Holstein–Primakoff boson operators [95], Bogoliubov transformation, and diagonalization. The Hamiltonian can consequently be expressed as

(11) \begin{equation*} {{\cal H}_{{\rm{exch}}}} = {E_{{\rm{cl}}}}\ + S\mathop \sum \limits_k {\omega _k} + \mathop \sum \limits_k 2S{\omega _k}\gamma _k^ + {\gamma _k},\end{equation*}

where *E*_cl_ is the classical ground-state energy, and the magnon energy can be found from $2S{\omega _k}$ [4,30,32,44,52,54,96]. To avoid needing a large unit cell to include the cycloid, local rotating coordinate systems were introduced both for BFO [4,52] and *o*-*R*MnO_3_ [34,35].

In BFO, the magnon modes were separated into two groups, according to two effective components of DMI: cyclon (Φ) and extra-cyclon (Ψ) modes, as shown in Fig. 2 [33]. Φ modes are gapless, while Ψ modes are gapped due to the pinning of the cycloid plane. Once the excitation spectrum has been obtained precisely, the Hamiltonian as well as the corresponding coupling constants can be evaluated precisely by fitting the excitation frequencies obtained by THz spectroscopy [5,8,47], Raman spectroscopy [5,6,14,45], IR spectroscopy [31] and INS [4,30,44]. Thereby, the best fitting results for *J* and *J*′ were found to be 4.38 meV and 0.15 meV, respectively [30,96]. A non-zero constant $\mathcal{K}$ will introduce cycloid deviation and splitting of the higher harmonics of the cycloid at every crossing of Φ_±n_ and Ψ_±n_ in BFO [52].

**Figure 3.**
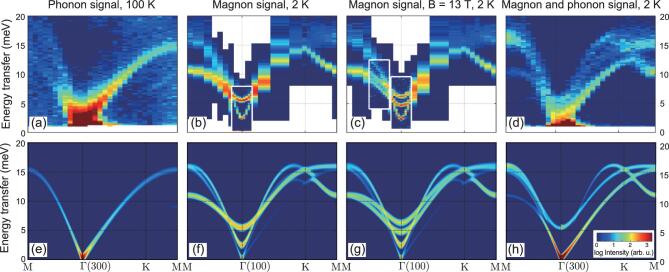
(a)–(d) Experimental magnon and phonon dispersions of *h*-YMnO_3_. (e)–(h) Theoretical dispersions with intensity at the same *q* paths, temperatures, and applied magnetic fields [43]. Copyright 2019, American Physical Society.

However, avoidance of crossing and linear splitting in both the *ab* plane and *c* direction exist in the magnon spectrum at the zone boundary and were observed in single-crystal YMnO_3_ INS

measurements, shown in Fig. 3. Therefore, the following lattice term ${{\cal H}_{\rm{L}}}$ and spin–lattice coupling term ${{\cal H}_{{\rm{SL}}}}$ [43,73] need to be considered in the Hamiltonian [97,98]:

(12) \begin{equation*} {\cal H}_{\rm {L}} = \mathop \sum \limits_{k,s} {\omega _{k,s}}a_{k,s}^ + \ {a_{k,s}} \end{equation*}

(13) \begin{equation*} {{\cal H}_{{\rm{SL}}}} = \sum_i {\sum_{\alpha \beta \gamma \delta } {{G_{\alpha \beta \gamma \delta }}S_i^\alpha S_i^\beta \epsilon _{\gamma \delta }^i} } ,\end{equation*}

where the bosonic operators *a_k_,_s_* with eigenenergies *ω_k_,_s_* are for acoustic phonons in a hexagonal lattice in the absence of magnetic order in *h*-YMnO_3_, and the spin–lattice coupling term ${{\cal H}_{{\rm{SL}}}}$ is a hybridization term between the Holstein–Primakoff magnon operators *S* and *G* [43]. This term denote the elastic energy introduced by the configuration *S^α^* and *S^β^*, where *G* is the magnetoelastic coupling tensor and *S* is the spin–phonon coupling tensor, the summation index *i* extends over the spins of the whole crystal.

The phonon–magnon interaction was further studied by using a quasiharmonic free-energy approach implemented using first-principles methods, where the coupling was regarded as the dependence of the exchange constant on the atomic displacements [87], although the anharmonic effect was not fully considered. Using this method, researchers found that the cycloid in BFO has a third harmonic, which was supported by THz spectroscopy measurements [5,8,31].

### Manipulation of quasiparticles

**Figure 4.**
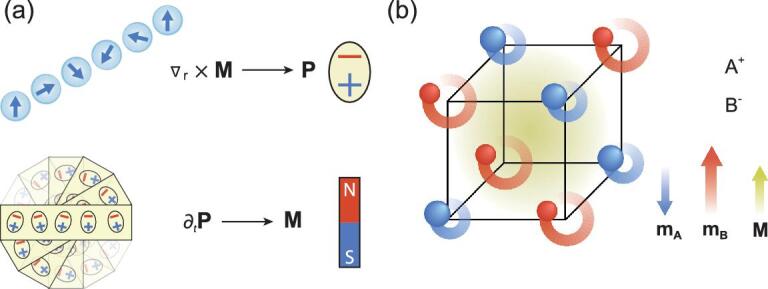
(a) Schematics of a polarization induced by a spatially varying magnetization and a magnetization induced by temporally varying polarization. (b) Magnetic moments from ionic loops. Perpendicular optical phonons drive ionic motion in a diatomic A^+^B^−^ material. Local magnetic moments, **m_A_** and **m_B_**, are created by the circular motions [100]. Copyright 2019, American Physical Society.

The strong interaction among the spin, polarization, and lattice in multiferroic materials provides plenty of space to manipulate the magnetization and polarization dynamically [99,100]. Previous researchers have put considerable effort into manipulating these features using strain [101–103]. In this article, an alternative method called ‘dynamic multiferroics’ will be reviewed. Magnetization *M* is symmetric under space inversion and antisymmetric under time reversal, whereas polarization *P* is symmetric under time reversal and antisymmetric under space inversion. Therefore, *M* ∼ *P* × ∂*P*/∂*t* can be developed in the presence of an appropriate dynamic polarization (Fig. 4).

This method supposes that $M \cdot ( {P \times {\rm{\ }}\partial P/\partial t} )$ couples *M* and *P* at the same order as $P \cdot ( {M \times ( {{\nabla _r} \times M} )} )$. The time variation of *P* can be realized by considering two adjacent *P* evolving with different frequencies *ω*_1_ and *ω*_2_. In this case, two intrinsic lattice vibrations with these frequencies contribute to different Born effective charges. Four possible coupling mechanisms have been proposed: (1) phonon Zeeman splitting in a magnetic field, with perpendicular polarized phonons with frequencies ${\omega _1} = {\omega _2} = {\omega _0}$; (2) resonant magnon excitation by optically driven phonons with ${\omega _1} \ne {\omega _2}$ with perpendicular polarity; (3) DM-type electromagnons, with an applied electric field and $P = ( {{P_1}( 0 ),{\rm{\ }}0,{\rm{\ }}{P_2}( t )} )$, where ${P_1} ( 0 ) = {P_{{\rm{FE}}}} ({{\omega _1} = 0,\varphi = \pi/2} )$ and ${P_2} ( t ) = E( t )({\omega _2} = {\omega _0})$, and (4) an inverse Faraday effect with perpendicular time-dependent polarizations induced by circularly polarized light both oscillating with the frequency of the light (${\omega _1} = {\omega _2}{\rm{\ }} = {\omega _0}$) and phase-shifted by *φ* = *π*/2. In multiferroic materials, each optical phonon is polarized with a certain polarization. Therefore, the above four schemes can be applied by carefully selecting activated phonons in a crystal.

**Figure 5.**
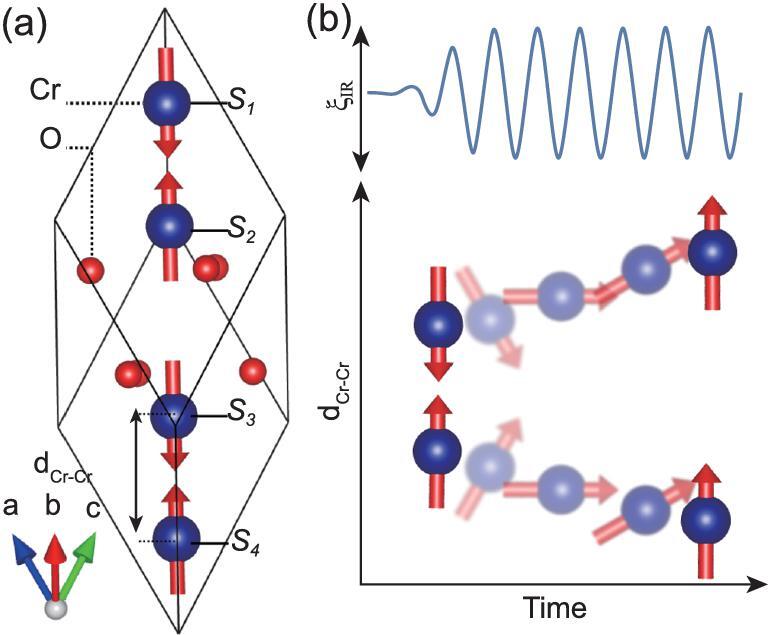
(a) Unit cell of Cr_2_O_3_. Red arrows represent the spin order. (b) Magnetic ground state changed by the excitation of a polar phonon mode [104]. Copyright 2019, American Physical Society.

Due to the recent development of THz technology, it is possible to activate phonons with frequencies in a certain range and certain polarizations. By combination with the strong coupling between the lattice and magnetic dynamics in multiferroic materials, it is thus possible to tune the magnetic states in these materials dynamically. The capabilities of this method were demonstrated by Fechner, as shown in Fig. 5 [104]. Fechner combined the well-established theory of non-linear phononics with spin dynamics, where the spin–lattice coupling was described through magnetic exchange interaction variations induced by structural changes and was demonstrated in three steps.

In the first step, the actual structural modification or atomic displacement induced via non-linear phononic coupling was evaluated by solving a set of dynamic equations, where an IR mode was excited by a driving sinusoidal force *F*(*t*) with a certain amplitude and frequency:

(14) \begin{eqnarray*} {\ddot{\xi }_{{\rm{IR}}}} + \omega _{{\rm{IR}}}^2{\xi _{{\rm{IR}}}} + {\gamma _{{\rm{IR}}}}\ \xi _{{\rm{IR}}}^3 = \ 2g{\xi _{{\rm{IR}}}}{\xi _{\rm{R}}} + F\!(t) \nonumber\\ \end{eqnarray*}

(15) \begin{equation*} {\ddot{\xi }_{\rm{R}}} + \omega _{\rm{R}}^2{\xi _{\rm{R}}} + {\gamma _{\rm{R}}}\ \xi _{\rm{R}}^3 = \ g\xi _{{\rm{IR}}}^2,\end{equation*}

(16) \begin{equation*} F\!\left( t \right) = {\rm{\ }}{E_{{\rm{drive}}}}\sin \!\left( {{\rm{\omega }}t} \right), \end{equation*}

where ${\xi _{\rm{R}}}$ and ${\xi _{{\rm{IR}}}}$ are the distortions of the Raman and IR modes, respectively; *γ*_IR_ and *γ*_R_ are the fourth-order anharmonic constants of the Raman and IR modes, respectively; and $g$ is the coupling between two phonon modes. Depending on the non-linear coupling between different modes, several phonon components can be excited by the driving modes.

In the second step, after obtaining certain variations of the phonon modes, the lattice–spin interaction was included to consider the position deviation of the magnetic exchange interactions between spins *i* and *j* to first and second order as

(17) \begin{eqnarray*} {H^{{\rm{sp}}}} &=& \sum_{\left\langle {i,j} \right\rangle } {\frac{{\partial {J_{i,j}}}}{{\partial \xi }}( {{{\boldsymbol{S}}_i} \cdot {{\boldsymbol{S}}_j}} )\xi} \nonumber\\ && +\, \mathop {\sum_{i,j} {\frac{{{\partial ^2}{J_{i,j}}}}{{\partial {\xi ^2}}}( {{{\boldsymbol{S}}_i} \cdot {{\boldsymbol{S}}_j}} ){\xi ^2}} }\limits_{} ,\end{eqnarray*}

where the exchange interaction can be expressed as a function of the mode amplitude as

(18) \begin{equation*} {J_{i,j}} ( {{\xi _{\rm{R}}}} ) = {J_{i,j}}\ + \frac{{\partial {J_{i,j}}}}{{\partial \xi }}{\xi _{\rm{R}}} + \frac{{{\partial ^2}{J_{i,j}}}}{{\partial {\xi ^2}}}\xi _{\rm{R}}^2 + \cdots . \end{equation*}

The last step was to consider the spin configuration evolution with respect to the time-dependent magnetic exchange modulations ${J_{i,\!j}}( {{\xi _{\rm{R}}}} )$ and the usage of classical magnetization dynamics by applying the LLG equation, with an atomic approach:

(19) \begin{eqnarray*}\frac{{d{S_i}}}{{dt}} &=& \ - \frac{\gamma }{{1 + {\alpha ^2}}}\left[ {{S_i} \times H_i^{{\rm{eff}}}\left( t \right)} \right] \nonumber\\ && -\, \frac{{\alpha \gamma }}{{1 + {\alpha ^2}}}\left[ {{S_i} \times \left[ {{S_i} \times H_i^{{\rm{eff}}}\left( t \right)} \right]} \right], \nonumber\\ \end{eqnarray*}

where $\alpha $ is the damping parameter for spins. The magnetic energy $H_i^{{\rm{eff}}}( t )$ was evolved by using a Heisenberg Hamiltonian only considering ${J_{i,\!j}}$ and ${D_{i,\!j}}$.

In this way, the magnetic state was tuned from the equilibrium AFM of Cr_2_O_3_ to AFM ordering with ferromagnetically coupled NN spins. This transition is driven by the change in NN magnetic exchange interaction when the Cr–Cr separation is modified through non-linear coupling of the optical phonons to a symmetry-conserving A_1g_ Raman-active mode.

## CONCLUSION

To achieve dynamic manipulation of magnetism as well as polarization, more detailed understanding of phonon–phonon and phonon–magnon interactions is required. Discrepancies still exist between the experimentally obtained dispersion curves and those theoretically predicted from first-principles or magnetic dynamics calculations. Moreover, demonstrations of the strong anharmonicity and illustrations of the ultrafast realistic evolution of these quasiparticles remain limited, either in terms of the resolution of the current characterization techniques or the capabilities of recent simulation methods. Despite these limitations, the strong coupling between phonons and magnons as well as electromagnons in AFM multiferroic materials makes it possible to apply the existing optical recording techniques for FMs to AFMs and to explore potential spintronic applications with ultrahigh speed and efficiency.
